# Transfemoral Approach in Revision Hip Arthroplasty: A Prospective Analysis of 36 Cases: Radiological and Functional Results at a Minimum 2 Years Follow-up

**DOI:** 10.3390/medicina58020237

**Published:** 2022-02-04

**Authors:** Vlad Alexandru Georgeanu, Tudor Atasiei, Vlad Predescu, Nicolae Gheorghiu, Andrei Marian Feier, Octav Marius Russu

**Affiliations:** 1 Department of General Medicine, “Carol Davila” University of Medicine and Pharmacy, 050474 Bucharest, Romania; tatasiei@yahoo.com (T.A.); ngheorghiu@hotmail.com (N.G.); 2 Clinic of Orthopaedics and Trauma Surgery, “St. Pantelimon” Hospital, 021659 Bucharest, Romania; 3 Ponderas Hospital, 021659 Bucharest, Romania; vpredes@pcnet.ro; 4 Department of Orthopaedics and Traumatology, Elias Emergency University Hospital, 011461 Bucharest, Romania; 5 Department of Orthopaedics and Traumatology, Clinical County Hospital, 540139 Tîrgu Mureș, Romania; andrei.feier@umfst.ro (A.M.F.); octav.russu@umfst.ro (O.M.R.); 6 Department of General Medicine, University of Medicine, Pharmacy, Sciences and Technology “George Emil Palade”, 540139 Tîrgu Mureș, Romania

**Keywords:** hip revision, stem removal, transfemoral approach, osteotomized bone fragment, hip stability

## Abstract

*Background and Objectives*: One of the most difficult aspects of hip revision is to remove the stem from the femoral canal with or without cement while maintaining the maximal amount of bone stock to obtain the best possible press-fit of the revision prosthesis. The transfemoral approach ensures direct access to the medullary canal so that the content removal is completed under direct control, while protecting the bone. This type of approach is particularly efficient for special conditions, such as deformation of the proximal femoral region, broken stems, or the presence of cement over a long distance distal to the prosthesis. The aim of this study was to evaluate the main advantages of transtrochanteric approach in hip revisions. *Materials and Methods*: Our series included 36 revisions performed using the transfemoral approach. We have analyzed the following postoperative radiological aspects: the length of the fixation zone distal to the osteotomized bone fragment (OBF), the gap between the OBF and the diaphysis, stem subsidence over time, and OBF consolidation. *Results*: The results were very good, both in terms of the rate of intraoperative complications and postoperative evolution. The fixation zone length was 4.2 cm on average (range: 2 to 5.8 cm). The distal gap between the OBF and the diaphysis was 1.2 cm on average (range: 0 to 2.3 cm). Stem subsidence was noted in four cases (11.1%). In all cases, stem subsidence occurred between three and six months and was 6 to 8 mm without affecting hip stability. OBF consolidation was radiologically confirmed for all cases at one year follow-up. Clinical assessment based on the Harris Hip Score showed an overall improvement from 43.2 preoperatively to 79.7 at 12 months and 83 at two years, respectively. The most important rate of progress was between 6 months and 12 months. *Conclusions*: The transtrochanteric approach has been shown to be very efficient for hip revisions. Understanding the hip biomechanics, applying a less aggressive surgical technique, and using efficient fixation methods such as cables significantly improved the results.

## 1. Introduction

In recent decades, hip arthroplasty has been one of the most successful orthopedic procedures and is practiced to an increasing degree. The revisions have multiplied significantly, as well as the degree of complexity, particularly their difficulty. An essential aspect of revision is the removal of the femoral component, with or without cement, which depends on the type of prosthesis and requires preservation of the bone stock. In some cases, this is difficult to achieve with an endofemoral approach, leading to either fractures or the sacrifice of a significant amount of bone, which has an unfavorable effect on the fixation of the revision stem. In septic conditions, the complete removal of any potentially contaminated material, such as cement and distal bone plug, increases the chances of infection healing. Other conditions, such as periprosthetic fracture, broken stems, and varus remodeling of the femur, are good indications for a transfemoral approach. This approach was popularized in the 1960s by Sir John Charnley, who used it for both primary arthroplasties and hip revisions, developing Ollier’s ideas from the previous century (1881). In 1999, Wagner proposed an extended lateral femoral osteotomy, using a transfemoral approach and osteotomizing half of the proximal femur. This method provided excellent results in cases of prosthetic revision [1]. Beginning in 1995, extended trochanteric osteotomy was proposed and popularized by Paprosky to simplify the access and removal of well-fixed cementless stems [2,3]. The major concerns of this approach are the consolidation of the osteotomy, displacement of the osteotomized bone fragment, stability of the hip itself, respectively, of the femoral stem. A careful surgical technique and avoiding as much trauma as possible both on the bone but especially on the soft tissues can produce excellent results, avoiding possible complications [4,5,6].

The aim of this study was to evaluate the main advantages of a transtrochanteric approach in hip revisions.

## 2. Materials and Methods

This is a prospective study reviewing 36 stem revisions that used the transfemoral approach and were performed between 2017 and 2019. The minimum follow-up was 24 months. Twenty-seven cases were total revision, and nine cases were stem revisions only. Twenty-two cases were cemented stems, and 14 were cementless. The initial diagnoses for hip replacement procedures were the following: hip arthritis (23 cases), rheumatoid arthritis (five cases), aseptic necrosis of the femoral head (five cases), and femoral neck fracture (three cases). Twenty-two patients were women, and 14 were men. The average age was 70.4 years (range 54–82 years). The BMI range was between 25.5 and 33.2. 

The study was conducted according to the guidelines of the Declaration of Helsinki and approved by the Ethics Committee of Emergency Hospital of Saint Pantelimon in Bucharest.

The indications for stem revision were the following: (a) 17 cases were stem loosening (47.2%), with varus remodeling of the proximal femur in four cases and a long cement plug distally to the stem tip in five cases; (b) six cases were periprosthetic joint infections, with a 22-month interval after the index procedure on average; one case was an early infection after revision (16.6%). For these cases, both the initial procedure (primary prosthesis removal, debridement, cement spacer) and revision were performed using a transfemoral approach; (c) four cases were Vancouver B2 periprosthetic fractures (11.2%); (d) seven cases were well-fixed stems associated with acetabular loosening, in which the revision was performed at both levels (19.4%); (e) two cases were broken stems (5.6%).

The cases were classified according to the Paprosky system for classifying femoral bone defects: type 1—two cases (5.5%); type 2—11 cases (30.5%); type 3A—15 cases (41.7%); type 3B—six cases (16.6%); type 4—two cases (5.5%). 

The prosthesis used was a modular curved conical cementless revision stem, Revitan (Zimmer, Warsaw, USA). The distal (diaphyseal) component was 2° tapered with longitudinal ribs, and a cover of Protasul^®^ titanium alloy. The proximal (metaphyseal) component was cylindrical or conical with dorsal and ventral ribs; the cylindrical component showed less prominent ribs in the sagittal plane, a slim design that prevented proximal jamming. The type was chosen depending on the aspect of the femoral metaphysis (in our series, 28 conical and 8 cylindrical). Thirty-two distal components were short (140 and 200 mm), with press-fit fixation at the level of the femoral isthmus, and four were long (260 mm), distally locked stems.

**Figure 1:**
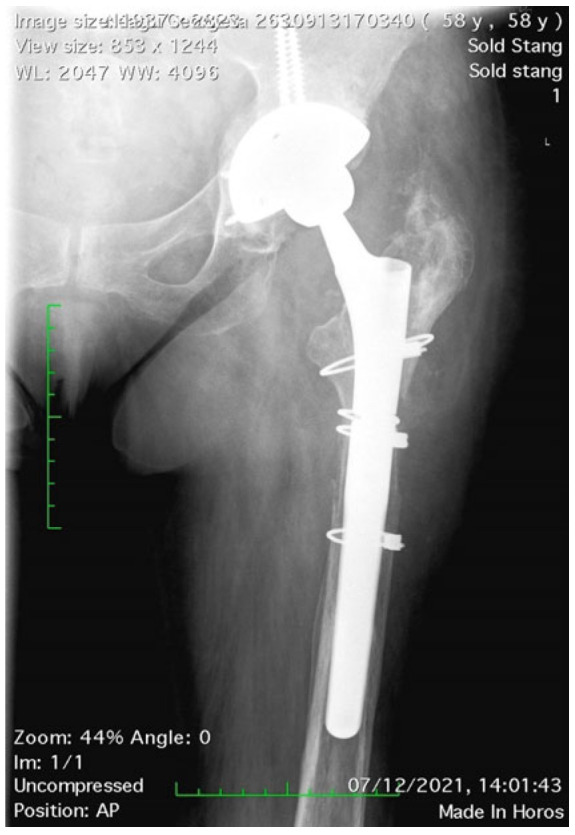
Maintaining the integrity of the femoral isthmus is mandatory; a minimal contact of 2 cm is necessary for primary stability of the stem.

**Figure 2:**
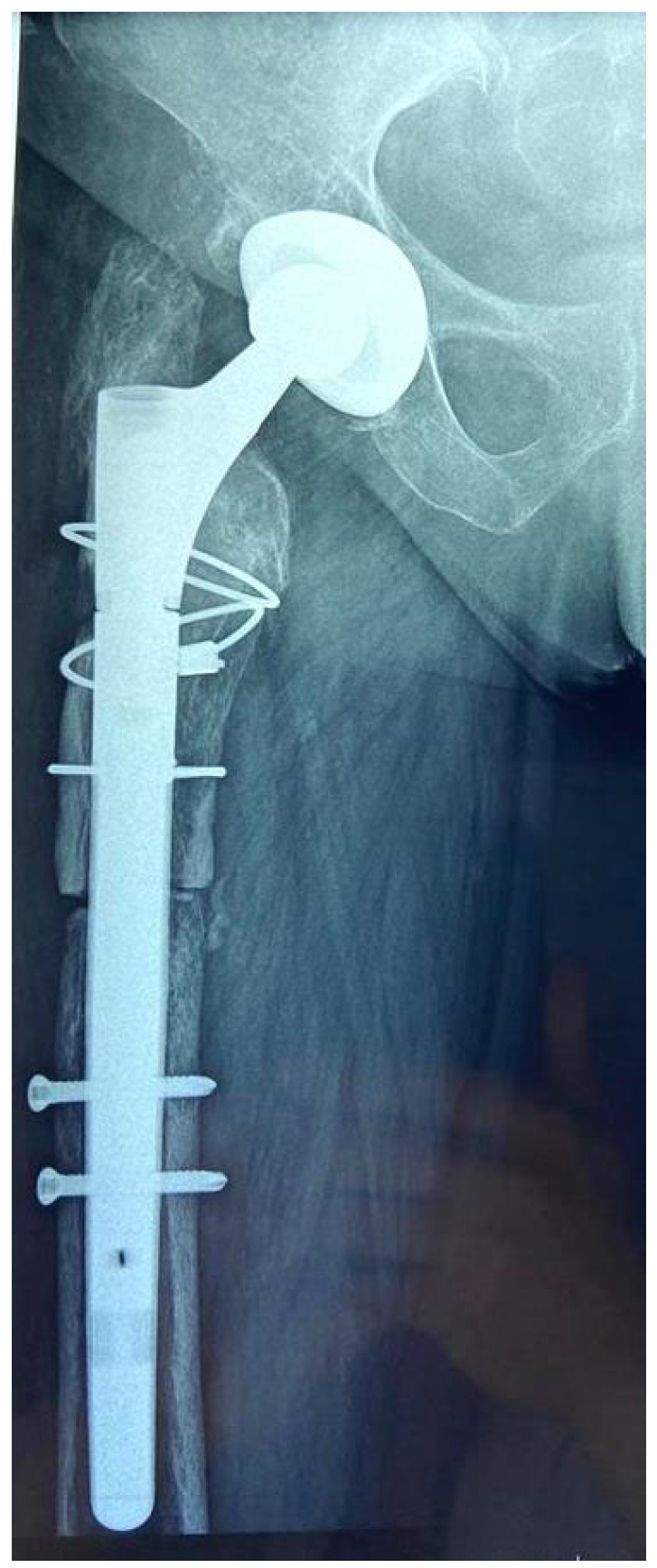
Internal cortex osteotomy for varus remodeling of proximal femur.

The length of the osteotomized bone fragment (OBF) is essential; it must be long enough to allow the easy removal of the stem with or without cement but without affecting the primary stability of the revision stem. Accurate preoperative planning is very important for this reason. On the right magnification A-P and profile hip X-rays, we measured the length of the stem and the length of the distal cement plug including the length of the plastic restrictor, taking the tip of the greater trochanter as a reference point. For cementless stems, it is not necessary for the OBF to reach the level of the stem tip, only up to approximately 2 cm, whenever it is preoperatively intact (Figure 1). Maintaining the integrity of at least the femoral isthmus is mandatory. Usually, the length of the OBF is 15–16 cm. A varus remodeling aspect can be corrected by adding the osteotomy of the internal cortex (Figure 2).

The posterior approach was used in all the cases. The vastus lateralis and its insertion on the OBF must be protected as much as possible while the vascular branches coming from the muscle are decisive in ensuring osteotomy consolidation [7]. At the distance established by preoperative planning, a transverse osteotomy was first made on the lateral aspect of the femur. The width of this osteotomy must be between 1/3 and 1/2 of the femoral circumference. A width smaller than one third will lead to difficulty approaching the medullary canal; a width larger than half will cause difficulty opening the OBF due to adhesion to the prosthesis. The length of the OBF in our series ranged between 12 and 19.5 cm, with a mean of 15.5 cm. The OBF was opened from the posterior to the anterior with the help of several wide chisels using the prosthesis as a fulcrum. Forcing the opening of the OBF can cause uncontrolled fractures of the OBF, the remaining bone cylinder, or of the greater trochanter. The OBF opening can be simplified by meticulous debridement of the fibrous tissue around the neck of the prosthesis and sectioning the rotators.

Removing the prosthesis and the cement from the medullary canal is usually easy; however, difficulties can be associated with removing a cementless long stem prosthesis, which is integrated distally to the OBF [8,9]. In addition, the opened osteotomy offers a good view of the interior of the femoral isthmus to prepare it. Implantation of the revision stem was performed with the OBF open, trying to obtain a good press distal to the OBF, especially at the level of the femoral isthmus. In four cases where the diaphyseal defect (Paprosky type 4) did not allow obtaining the press-fit at this level, a long stem was used, which was distally locked with two screws.

Adapting the shape of the trochanteric region of the OBF to the lateral profile of the prosthesis had to be performed very carefully using thorough debridement. The OBF was fixed using two or three Dall–Miles cables, adequately tensioned, depending on the length. No difference was found between two- and three-cable fixation in terms of the peak force, stiffness of the construction, or angular or axial displacement of the osteotomy [10,11]. Recently, numerous other techniques for fixing the OBF have been described using either metal wires or cables arranged in different configurations or synthetic non-metallic materials. In all these variants, the reported results were good [12,13,14].

Fixation of periprosthetic fractures was performed in all four cases in our series using Dall–Miles plates with distal screws and proximal cables after exchanging the primary prosthesis with a long revision stem which bypassed the fracture. This internal fixation method for periprosthetic fractures has been shown to be efficient in many biomechanical studies [15].

## 3. Results

The surgical time was 210 min on average with a range between 150 and 320 min. The average perioperative blood loss (intraoperative and first 24 h) was 450 mL (200–1250 mL), mainly occurring intraoperatively. The amount of blood loss decreased significantly with the use of IV tranexamic acid on a regular basis.

The intraoperative complications were the following: five fractures of the OBF (13.8%) that did not require an additional fixation apart from three Dall–Miles cables, usually used for OBF fixation; three fractures of the trochanter (8.3%) that required fixation with wire (two cases); a trochanter plate (one case); and one undisplaced fracture of the femoral shaft, distal to the OBF (2.8%) fixed with another Dall–Miles cable. The most common intraoperative complication, OBF fracture, occurred in most cases due to mishandling the fragment during surgery and, much less frequently, during osteotomy. The general percentage of femur fractures was similar to the incidence reported in previous studies in the literature [16,17].

The X-ray postoperative protocol followed four aspects: length of the fixation (anchorage) zone, distal to the OBF; the gap between the osteotomy and the diaphysis; stem subsidence over time; and OBF consolidation.

Theoretically, primary stability by press-fit in the diaphysis must be achieved on a 5 cm length. When a good press-fit is hard to obtain, especially in Paprosky type 4 bone defects, the fixation of the stem must be supplemented by distal locking with two or three screws. Among the 36 cases of our study, we used distal locking in four cases (two for type 3B and two for type 4). For the other 32 cases, the fixation zone was 4.2 cm on average (ranging between 2 and 5.8 cm).

The distal gap between the OBF and the diaphysis was 1.2 cm on average (range: 0 to 2.3 cm). The distance had not increased in any of the cases at subsequent radiological follows-up, which demonstrated the solidity of the fixation of the OBF with cables. At one year, this space disappeared in all cases with an initial length of less than 1.5 cm (20 cases). For the gaps larger than 1.5 cm, complete filling was noted in 10 out of 16 cases. In the other six cases, partial filling was obtained but without any functional effect.

Stem subsidence was noted in four cases (11.1%). In all cases, stem subsidence occurred between three and six months. The defect type was Paprosky 2 B in two cases, 3A in one case, and 3B in one case. For cases with a 2B defect, the fixation area was 4.8, respectively, 5.2 cm in length. However, the stem diameter was too small, which caused a subsidence of 8, respectively, 6 mm. For both the 3A and 3B defect type cases, the subsidence was 8 mm. Subsidence did not lead to the dislocation of the prosthesis in either of the cases. For the remaining cases, subsidence was not noted, independently to the length of the fixation zone or the type of bone defect, even for the case with an undisplaced diaphyseal fracture fixed with a cable.

OBF consolidation was radiologically confirmed for all cases at one-year follow-up, despite delayed consolidation noted at six months after surgery for nine cases.

The clinical evolution was determined using the Harris hip score. All the statistical analysis was performed in Python version 3.7.12 using the libraries NumPy (version 1.19.5) and Matplotlib (version 3.2.2).

As expected, regardless of the type of bone defect, the Harris score showed continuous improvement in successive examinations at 3, 6, 12, and 24 months. Younger patients had the best preoperative scores, regardless of the type of defect, respectively, the cases with a lower degree of bone defects. The same cases showed a faster improvement of the Harris score. The lowest preoperative score and the lowest rate of improvement in the Harris score was observed in elderly patients and patients with major bone defects (3 and 4 Paprosky). In these cases, there were no differences between the cases in which the fixation was press-fit and cases where distal locking was used (Table 1).

The statistical comparison of the Harrison Hip Score between the preoperative period and all follow-up periods showed statistically significant differences (*p* < 0.005).

**Figure 3:**
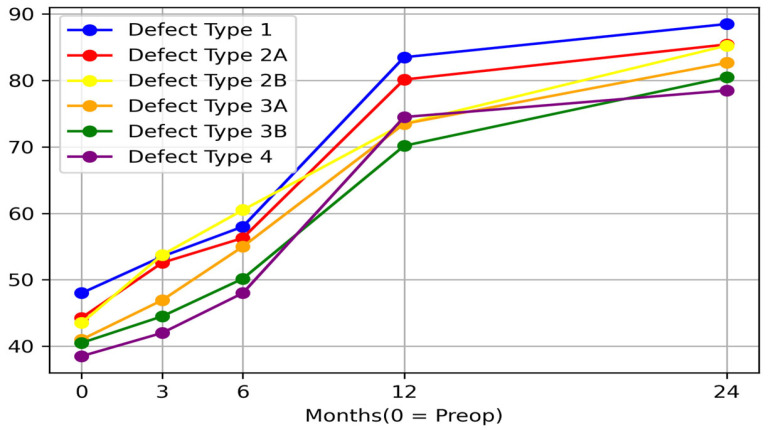
Time evolution for Harris Hip Score for all defects. Values are expressed as arithmetic mean; 0 is considered preoperative period.

The highest rate of progress was registered in the interval between 6 and 12 months. Despite the long interval between 12 and 24 months follow-up, progress was not as important as in the previous interval (Figure 3).

## 4. Discussion

The differences between the extended trochanteric osteotomy and the transfemoral approach consist in the width and length of the OBF. For an extended trochanteric osteotomy, the OBF is approximately a third of the femoral circumference, with an average length between 12 and 14 cm; the transfemoral approach is defined by a larger OBF that is half the width of the femoral shaft circumference and longer than 15 cm; distally, the osteotomy is extended immediately above the isthmus of the femur or to the apex of the deformity [18,19]. Starting from this aspect, it is difficult to precisely define the type of approach, since the length of the OBF is adapted to the particular aspects of the cases. Both variants are mechanically and biologically more efficient than the traditional transtrochanteric approach, described by Charnley. The longer the osteotomized fragment, the easier the bone healing is due to the larger contact surface between the bone fragments [20]. Mechanically, a longer OBF keeps a larger insertion of the vastus lateralis intact, effectively balancing the action of the hip abductors, especially the gluteus medius, thus reducing the proximal and anterior displacement of the OBF [21,22].

The indications for the transfemoral approach are currently very large: a loosened cemented stem with areas of well-fixed cement mantle; long cement content of the femoral canal, distal to the stem tip; difficult removal of an uncemented stem due to the remnant integration areas of the stem; remodeling in varus of the proximal femoral part; greater trochanter malposition or the trochanteric region deformity after other procedures such as osteotomies or fractures; Vancouver B2 or B3 periprosthetic fractures; thin cortical bone prone to the fractures; broken stem. A special indication is a difficult approach of the acetabulum.

This approach is very efficient for removing the primary femoral component, cementless or cemented, with the cement mantle and the distal restrictor. In addition, the visualization of the medullary canal is very good, ensuring the removal of the entire amount. The thorough removal of cement and distal restrictor is critical, especially in septic cases. Any foreign, possibly contaminated material decreases the chances of eradicating the infection, even with antibiotic treatment. In periprosthetic joint infections, the transtrochanteric approach can be used for both one- and two-stage revisions. Our positive results in these septic cases were confirmed by data from literature that showed a 100% union rate of the extended trochanteric osteotomy (ETO), confirmed during the second-stage procedure [23,24], and a successful eradication of infection in 96% of cases.

The presence of remnant cement fragments can deflect the tools used for preparing the medullary canal (reamers, rasps) or even of the revision stems, leading to perforations or fractures of the diaphyseal cortex.

Another issue related to the presence of cement inside the medullary canal is the integration of the cementless revision stem by limiting the contact surface with the bone as well as under-sizing the stem. Thoroughly removing the cement also improves the bone ingrowth of the revision stem. Bone ingrowth is also promoted by the creation of the OBF itself; it has the same healing mechanism as a fracture since it is actually a controlled fracture. The reduction of this controlled fracture of the stem and the close apposition of the bone fragments induces much wider contact between the bone and the prosthesis than in an endofemoral approach, where three points of contact are used [8].

In 30% of stem loosening cases, varus remodeling of the metaphyseal–diaphyseal area of the femur has been observed [9]. In these cases, the transtrochanteric approach is associated with an osteotomy of the medial cortex to correct the varus deformity, thus avoiding the perforation or the fracture of the femur while preparing the medullary canal and inserting the stem. In our study, four cases presented with significant remodeling in varus that required osteotomy of the internal cortex; in all cases, we obtained correction of the diaphyseal axis and consolidation of the osteotomy [25]. 

Typically, the femoral OBF can be reduced so that the gap between the OBF and the diaphysis is minimal. However, this kind of reduction cannot always be achieved, maintaining a significant distance which fills with bone in the healing process. From this practical aspect, the proximal or distal displacement of the OBF to correct the tension in the abductors is quite easy to achieve, and the results are very good [26].

The displacement of the femoral OBF provides excellent visualization of the acetabulum and the posterior column, which is beneficial for reconstructing it. Good visualization is also useful in cases where the removal of the acetabular component is difficult either due to intrapelvic displacement or when it is not possible to disengage the prosthetic components, such as in hemiarthroplasties. In these situations, attempting to dislocate the prosthesis can cause fractures of either the acetabulum or the femur. The transtrochanteric approach ensures easy removal of the acetabular component without creating additional tension in the bone [27,28,29].

An important indication of the transtrochanteric approach is periprosthetic fractures. Vancouver type B2 and B3 fractures, in which the fracture is near or just distal to the loose stem (B2) or loosely associated with a poor bone stock (B3), are suitable for this kind of approach. Protection of the bone is the most important aspect in stem removal, especially for B3 type fractures when the bone is very thin and fragile [30]. In our study, this type of fracture showed a very good evolution of the healing of both the fracture and the OBF; in all cases, only cables were used to fix them, and it was not necessary to supplement the fixation with plates. These good results were confirmed by data from the literature reporting 100% fracture and OBF healing [31,32,33].

The non-union rate of the osteotomy is very low (0–4.4%) in the last period of time [34,35]. Data from the literature have confirmed that maintaining the best possible vascularization of the OBF with minimal disinsertion of the vastus lateralis from the OBF is essential for this. In addition, the best possible reduction of the osteotomy, thus providing a large surface area of bone contact, as well as firm fixation, are other elements that can improve the chances of bone healing.

## 5. Conclusions

The transtrochanteric approach has been shown to be very efficient for hip revisions. Understanding the hip biomechanics, applying a less aggressive surgical technique, and using efficient fixation methods such as cables significantly improved the results. Despite seeming to be a more aggressive procedure, the transfemoral approach is a relatively easy procedure that makes it possible to remove the prosthesis quickly and clean the medullary canal. The newly formed bone forms within several months after surgery.

The main advantage of this method is the ease of stem removal, both of a cemented prosthesis but also a well-integrated cementless prosthesis especially. Cement removal is also much easier than in an endofemoral approach, thus reducing both the operating time and possible complications such as perforation or fracture of the femur; the complete removal of the cement is mandatory for septic revisions. Periprosthetic fractures, especially Vancouver types B2 and B3, can be effectively treated using this type of approach. The visualization of the acetabulum and the correct tension of the abductors by repositioning the trochanter are other advantages of this method.

The excellent results obtained in terms of the reduction in the operative time, lower rate of complications, and postoperative functional recovery make this method the method of choice for us in most hip revisions.

## Figures and Tables

**Table 1:** Evolution of Harris Hip Score for each type of bone defect according to Paprosky classification; values are expressed as arithmetic mean +/− standard deviation.

Type of Defect	Number of Cases	Harris Hip Score				
		Preoperative	3 Months	6 Months	12 Months	24 Months
1	2	48.00 ± 1.41	53.50 ± 0.71	58.00 ± 1.41	83.50 ± 2.12	88.50 ± 2.12
2A	7	44.28 ± 1.88	52.57 ± 1.71	56.28 ± 2.28	80.14 ± 1.51	85.42 ± 2.93
2B	4	43.50 ± 2.51	53.75 ± 2.98	60.50 ± 6.02	73.50 ± 3.41	85.25 ± 1.50
3A	15	41.00 ± 1.81	46.93 ± 2.71	55.00 ± 3.62	73.46 ± 2.61	82.66 ± 2.60
3B	6	40.50 ± 1.64	44.50 ± 3.08	50.16 ± 3.18	70.16 ± 2.71	80.50 ± 1.87
4	2	38.50 ± 0.70	42.00 ± 0.00	48.00 ± 0.00	74.50 ± 0.70	78.50 ± 0.70
All	36	42.08 ± 2.79	48.47 ± 4.43	54.83 ± 4.67	74.83 ± 4.47	83.22 ± 3.27
